# Prior Concussions and Risk of Disability for Patients After a Motor Vehicle Crash

**DOI:** 10.1001/jamanetworkopen.2025.54831

**Published:** 2026-01-21

**Authors:** Donald A. Redelmeier, Vidhi Bhatt, Samantha S. M. Drover

**Affiliations:** 1 Evaluative Clinical Sciences Program, Sunnybrook Research Institute, Toronto, Ontario; 2 Institute for Clinical Evaluative Sciences, Toronto, Ontario; 3 Department of Medicine, University of Toronto, Toronto, Ontario; 4 Division of General Internal Medicine, Sunnybrook Health Sciences Centre, Toronto, Ontario; 5 Center for Leading Injury Prevention Practice Education & Research, Toronto, Ontario

## Abstract

**Importance:**

Recovery after a motor vehicle crash can be slow, frustrating, and incomplete with lingering neurocognitive complications.

**Objective:**

To examine whether a prior concussion is associated with increased risk of long-term disability after a motor vehicle crash.

**Design, Setting, and Participants:**

This cohort study included adults surviving a motor vehicle crash treated in Ontario, Canada, from April 2003 to March 2023 with analyses to November 2025.

**Exposure:**

Prior concussion.

**Main Outcome and Measures:**

Long-term disability defined by official social service records, comparing those with a prior concussion and those with no history of a prior concussion.

**Results:**

A total of 907 984 patients injured in a motor vehicle crash were included (mean [SD] age, 37 [14] years; 472 435 male [52.0%]); 19 851 patients had a prior concussion and 888 133 had no prior concussion. A total of 54 678 patients were subsequently diagnosed with long-term disability over 9 543 505 patient-years of follow-up (mean [SD], 10.5 [5.8] years), equal to an absolute risk of 1 in 17 per decade (5.7 per 1000 patient-years). Patients with a prior concussion had a 15% higher adjusted risk of long-term disability (95% CI, 9%-21%; *P* < .001) compared with those with no prior concussion. The increased risk of long-term disability was independent of other measured risk factors, applied to diverse patient groups, and included those involved as passengers or pedestrians. The increased risk extended across a spectrum of crash severity, was accentuated for single-vehicle events, replicated in analyses with artificial intelligence models adjusting for confounding, and remained distinct from the risks of death, readmission, or short-term health care costs.

**Conclusions and Relevance:**

This population-based cohort study suggests a significant increased risk of long-term disability after a motor vehicle crash, particular among patients with a prior concussion. More efforts at concussion prevention and traffic safety counseling may be justified for patients.

## Key Points

**Question:**

Is a prior concussion associated with an increased risk of long-term disability following a motor vehicle crash?

**Findings:**

In this cohort study of 907 984 adult patients, a prior concussion was associated with a 15% increased risk of long-term disability.

**Meaning:**

This finding suggests the importance of counseling patients about the risks of motor vehicle crashes and previous concussions, as well as the importance of follow-up care to reduce the risk of subsequent disability.

## Teaser

This cohort study of Canadian adults surviving a motor vehicle crash examines the association of prior concussions with risk of long-term disability.

## Introduction

A concussion is an acute head injury caused by energy that compromises brain function temporarily. Research suggests a prior concussion may contribute to subsequent motor vehicle crash risks for patients.^1,2^ Whether a concussion might also limit neurocognitive reserve and lessen recovery after a later injury is unknown, especially in comparison to other factors that influence recovery.^3,4^ On the one hand, a prior concussion might lead to neurocognitive complications and residual impairments that limit a patient’s health.^5,6^ On the other hand, a prior concussion may be a trivial factor compared with a patient’s full profile of environment, lifestyle, and personal risks.^7,8,9^

Motor vehicle crashes are a common health threat with a 30% to 70% lifetime risk for the average individual in the US.^10^ Most people do not die from a crash, yet many do not recover completely and some have a variable course with lasting neurocognitive complications.^3^ In addition, people remember a crash and may make behavioral changes to compensate afterwards. A greater awareness of long-term prognosis might help in targeting symptomatic interventions for patient recovery and prevention. A rigorous estimate about the future can also help reconcile mistaken expectations, reduce conflicts about public health planning, and potentially aid in counseling patients about traffic safety.^11^

However, previous studies provide little information on long-term recovery after a motor vehicle crash. Instead, the prevailing trauma research tends to focus on a patient’s short-term physiology rather than long-term disability.^12^ Risk factors contributing to a serious crash are not always the same as prognostic factors that determine recovery.^13^ The purpose of this study was to explore directly whether prior concussion is associated with how well a patient recovers from a subsequent motor vehicle crash. We hypothesized that individuals with prior concussion might have increased risk of disability after a motor vehicle crash occurring years later.

## Methods

### Study Setting

We conducted a population-based cohort analysis in Ontario, Canada’s most populated region, with 10 980 900 individuals in 2013 (study midpoint), 8 920 342 registered motor vehicles, and 60 088 motor vehicle crashes causing injury.^14^ Disability support programs were available for adults who qualified for income support due to chronic disabilities (population prevalence 41 per 1000 adults, annual incidence 3 per 1000 adults, lifetime costs averaging $200 000 per adult).^15,16,17,18^ Universal health insurance guaranteed access to emergency care and electronic records were available for authorized investigators to conduct population-based health research. The Institute for Clinical Evaluative Sciences (ICES) is an independent, nonprofit research institute that analyzes health care data for health system evaluation and improvement.^19,20^

ICES is a prescribed entity under Ontario’s Personal Health Information Protection Act (PHIPA). Section 45 of PHIPA authorizes ICES to collect personal health information, without consent, for the purpose of analysis or for compiling statistical information with respect to the management of, evaluation or monitoring of, the allocation of resources to, or planning for all or part of the health system. Projects that use data collected by ICES under section 45 of PHIPA, and use no other data, are exempt from institutional review board approval. The use of the data in this project was authorized under section 45 and approved by ICES’ Privacy and Legal Office. The reporting of this study results followed the Strengthening the Reporting of Observational Studies in Epidemiology (STROBE) reporting guideline.

### Motor Vehicle Crash

We identified patients injured in a motor vehicle crash throughout the region (178 emergency departments) during a 20-year interval (April 1, 2003, to March 31, 2023). We excluded children (age 17 years or younger), seniors (age 65 years or older), those with a home address outside Ontario, and those with preexisting disability (to ensure each patient was eligible for subsequent disability support). These methods (eAppendix 1 in Supplement 1) have been validated in past research.^21,22,23^ Individuals with more than 1 crash were analyzed according to first incident so that each patient was included once in analysis. Additional crash details included timing (hour), configuration (single, multiple vehicles), position (driver, passenger, pedestrian), need for ambulance (yes, no), triage severity (Canadian Triage and Acuity Scale [CTAS] score), hospitalization (yes, no), and initial trauma assessment (Injury Severity Scale).^24^

### Prior Concussions

We identified a prior concussion diagnosis by accessing physician billing data from earlier years using linkage algorithms and encoded identifiers (eAppendix 2 in Supplement 1) based on the *International Classification of Diseases, Ninth Revision (ICD-9)* diagnosis (code: 850).^25^ This diagnostic code for concussion has been validated with excellent specificity (99%) and moderate sensitivity (46% to 76%).^26,27^ The look-back interval spanned 5 years prior to the crash to consistently count all patients in accord with the time of inception of databases. Additional details included time from most recent concussion (2 years or longer, within 2 years) and number of separate concussions (single vs multiple). The available databases lacked information on Rivermead Post-Concussion scores, the Sport Concussion Assessment Tool, or other measures for gauging concussion severity.^28,29^

### Additional Characteristics

Further patient characteristics were obtained by computerized linkages to multiple health care records using validated algorithms and unique patient identifiers (eAppendix 2 in Supplement 1).^30^ The demographic registry was used to determine the patient’s age (years), sex (binary), socioeconomic status (quintile), and home location (urban, rural).^31,32^ The physician services database provided data on clinic visits, emergency contacts, and hospitalizations to collect diagnoses before the motor vehicle crash.^33^ Specific attention was directed to demographic factors associated with long-term disability (eg, low socioeconomic status) and diagnoses associated with long-term disability (eg, alcohol misuse). The available databases contained no information on social factors that influence employment including training, salary, family supports, or work satisfaction.^34,35^

### Subsequent Disability

Subsequent long-term disability was defined by the submission of a formal disability support application as identified by official social service records (OHIP codes: K050-K054, K057-K060). Disability applications in this setting required medical reports from a physician (Activities of Daily Living Index, Health Status Report, Special Necessities Benefit Form). This approach to identifying long-term disability has been validated in past research yet may underestimate total disability.^36,37^ The available databases did not contain information on the nature of disability, how findings were authenticated by a physician, or whether an application was eventually denied. Secondary analyses considered alternative long-term outcomes including all-cause mortality, hospital readmission for any reason, and subsequent health care costs over 1 year.

### Statistical Analysis

The primary analysis examined the risk of long-term disability after a motor vehicle crash and compared patients with a prior concussion relative to those without a prior concussion. We defined the follow-up interval as starting on the day of hospital discharge and included only individuals who survived acute injuries. We used unadjusted cumulative incidence curves to evaluate survivors for death or disability during follow-up, with 2 years as the predefined landmark for estimating absolute risks (censoring anomalous empty records after 5 years). Relative risks were estimated based on the proportional hazards model before and after adjusting for baseline characteristics (model structured by subdistributional hazard ratios; model fit assessed by C-index).^38,39^ Patients not known to be dead or disabled were assumed alive and healthy.

Secondary analyses were conducted to further explore the robustness of results and relevance to specific patient groups. The time profile was tested by examining alternative landmarks aside from 2 years (1, 5, 10 years). Generalizability was tested by reexamining associations after stratifying on specific crash details (timing, position, configuration, severity). Confounding was assessed by applying Artificial Intelligence methods (XGBoost) as well as a separate propensity score analysis as further tests of the robustness of results (eAppendix 3 and eTable 3 in Supplement 1). A basic dose-response association was tested by assessing risk of long-term disability for those with a single concussion compared with multiple prior concussions. XGBoost statistical analyses were conducted using R software (version 3.6.1; package 1.4.0.1) (R Project for Statistical Computing) and all other analyses using SAS Enterprise Guide version 8.3 (SAS Institute Inc). A 2-sided *P* < .05 was specified as the threshold for statistical significance throughout.

## Results

### Baseline Characteristics

A total of 907 984 patients were injured in a motor vehicle crash and required emergency medical care (mean [SD] age, 37 [14] years; 472 435 male [52.0%]), of whom 19 851 had a prior concussion and 888 133 had no prior concussion (eFigure in Supplement 1). The 2 groups spanned a diverse range of demographic characteristics, medical diagnoses, and socioeconomic status (Table 1). The largest relative differences were that patients with a prior concussion tended to be younger (ages 18 to 39 years: prior concussion, 76.5% [15 851 of 19 851 patients] vs 57.8% [513 565 of 888 133 patients]), more likely to have a history of alcohol misuse (1.5% [303 of 19 851] vs 0.8% [6952 of 888 133]), and more likely to have a mental health diagnosis (eg, anxiety: 25.1% [4979 of 19 851] vs 15.7% [139 607 of 888 133]). Other diseases showed smaller differences or were less frequent among patients with prior concussion, including diabetes, osteoarthritis, or hypertension. Common causes of mortality, including heart disease and cancer, were rare and observed in fewer than 5% of patients in both groups.

**Table 1.** Baseline Patient Characteristics

Characteristics	Patients, No. (%)	
	Prior concussion (n = 19 851)	No prior concussion (n = 888 133)
Demographic data		
Age, y		
18-39	15 185 (76.5)	513 565 (57.8)
40-65	4666 (23.5)	374 568 (42.2)
Sex		
Male	9889 (49.8)	462 546 (52.1)
Female	9962 (50.2)	425 587 (47.9)
Home location		
Urban	16 891 (85.1)	781 009 (87.9)
Rural	2960 (14.9)	107 124 (12.1)
Past diagnoses^a^		
Alcohol misuse	303 (1.5)	6952 (0.8)
Diabetes	578 (2.9)	42 350 (4.8)
Hypertension	764 (3.8)	59 760 (6.7)
Heart disease	668 (3.4)	30 433 (3.4)
Syncope	2350 (11.8)	54 459 (6.1)
Sleep apnea	1232 (6.2)	42 525 (4.8)
Osteoarthritis	525 (2.6)	28 904 (3.3)
Depression	1395 (7.0)	31 288 (3.5)
Anxiety	4979 (25.1)	139 607 (15.7)
Cancer	464 (2.3)	23 544 (2.7)
Socioeconomic status quintile^b^		
Highest	4072 (20.5)	156 359 (17.6)
Next highest	4142 (20.9)	177 179 (19.9)
Middle	3920 (19.7)	182 936 (20.6)
Next lowest	3842 (19.4)	184 650 (20.8)
Lowest	3875 (19.5)	187 009 (21.1)

^a^ Based on previous year.

^b^ Based on home neighborhood, missing data coded as lowest.

### Acute Care

Motor vehicle crash patterns were comparable for the 2 groups as characterized by incident time, vehicle configuration, and individual position (Table 2). Initial mean (SD) injury severity scores were similar for patients with a prior concussion (2.65 [3.84]) compared with those without a prior concussion (2.35 [3.75]). Initial triage severity also showed no significant imbalance between the 2 groups. Over a third required emergency ambulance transport, with a significantly lower frequency for patients with a prior concussion relative to those without a prior concussion (38.9% [7724 of 19 851] vs 43.9% [390 189 of 888 133]). Rates of hospital admission were significantly lower for patients with a prior concussion (3.0% [603 of 19 851] vs 4.2% [36 991 of 888 133]). In addition, mean (SD) length of stay among those admitted was shorter for patients with a prior concussion (7.83 [10.43] days) than those without a prior concussion (9.49 [18.91] days).

**Table 2.** Traffic Crash Patterns

Characteristics	Prior concussion	
	Yes (n = 19 851)	No (n = 888 133)
Crash features		
Time^a^		
Dawn	871 (4.4)	42 819 (4.8)
Morning	3490 (17.6)	167 386 (18.8)
Afternoon	4739 (23.9)	211 897 (23.9)
Evening	5568 (28.0)	249 577 (28.1)
Night	3732 (18.8)	161 488 (18.2)
Late night	1451 (7.3)	54 966 (6.2)
Configuration^b^		
Single vehicle	5883 (29.6)	237 119 (26.7)
Multivehicle	10 218 (51.5)	479 682 (54.0)
Not motorized	2720 (13.7)	126 273 (14.2)
Unlisted	1030 (5.2)	45 059 (5.1)
Position^c^		
Driver	10 478 (52.8)	474 066 (53.4)
Passenger	4736 (23.9)	200 314 (22.6)
Pedestrian	4637 (23.4)	213 753 (24.1)
Emergency care^d^		
Ambulance	7724 (38.9)	390 189 (43.9)
High triage severity	4630 (23.3)	188 287 (21.2)
Hospital admission	603 (3.0)	36 991 (4.2)
Injury Severity, mean (SD)^e^	2.65 (3.84)	2.35 (3.75)
Days in Hospital, mean (SD)^f^	7.83 (10.43)	9.49 (18.91)

^a^ Boundaries for time period were 4:00 am, 8:00 am, 12:00 pm, 4:00 pm, 8:00 pm, and 12:00 am.

^b^ *International Statistical Classification of Diseases and Related Health Problems, Tenth Revision (ICD-10)* codes single and multiple vehicle events.

^c^ Pedestrian includes bicycles, scooters, and other nonprotected road users.

^d^ Assessed in emergency department for traffic crash.

^e^ *ICD-10* algorithm; range, 0 to 75; higher indicates worse injury.

^f^ Based on cases admitted.

### Subsequent Disability

**Figure 1.**
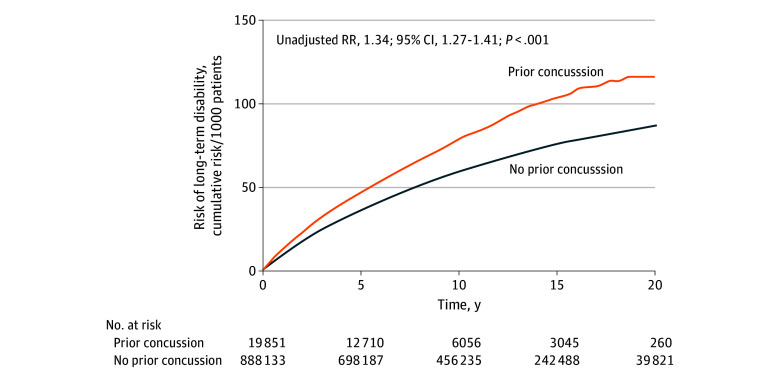
Risk of Long-Term Disability Cumulative incidence plots of unadjusted absolute risk of long-term disability after a traffic crash. Results show increased risk of long-term disability for patients with a prior concussion. For context, population norm is 3 per 1000 patients annually (equal to 6 per 1000 patients after 2 years and 60 per 1000 patients after 20 years).

A total of 54 678 individuals developed a disability during follow-up, equivalent to an average rate of 5.7 per 1000 person-years. Patients with a prior concussion accounted for 1311 cases of disability over 160 787 patient-years, equal to an absolute rate of 8.2 per 1000 annually. Patients without a prior concussion accounted for 53 367 cases of disability over 9 382 718 patient-years, equal to an absolute rate of 5.7 per 1000 annually. The difference corresponded to a 34% relative increased risk of long-term disability associated with a prior concussion (95% CI, 27%-41%; *P* < .001). The increased risk became apparent a few months after injury, persisted over extended years of follow-up, and equaled 1 case of long-term disability for every 180 survivors at the 2-year landmark (Figure 1).

#### Additional Risk Factors

The increased risk of long-term disability after a motor vehicle crash was associated with many other individual characteristics. In particular, younger age (relative risk [RR], 1.18; 95% CI, 1.16-1.21), male sex (RR, 1.13; 95% CI, 1.11-1.15), a rural home location (RR, 1.09; 95% CI, 1.07-1.12), and lower socioeconomic status (lowest quartile: RR, 1.94; 95% CI, 1.89-1.99) were each associated with an increased risk of long-term disability in adjusted analyses (Table 3). As expected, a diagnosis of alcohol misuse was a substantial risk factor (RR, 2.70; 95% CI, 2.56-2.84). A diagnosis of depression (RR, 1.78; 95% CI, 1.72-1.84), anxiety (RR, 2.00; 95% CI, 1.96-2.04), diabetes (RR, 1.48; 95% CI, 1.42-1.53), and hospital admission (RR, 1.10; 95% CI, 1.06-1.15) were additional risk factors to lesser degrees. Conversely, diagnoses of treated hypertension or cancer were not significant risks. The model fit was mediocre (*C* statistic = 0.684 overall, 0.722 at 2-year landmark). Adjusting for all measured characteristics showed a 15% relative increased risk of long-term disability for patients with a prior concussion (95% CI, 9%-21%; *P* < .001).

**Table 3.** Factors Associated With Long-Term Disability

Characteristics	Relative risk (95% CI)^a^	
	Basic analysis^b^	Adjusted analysis^c^
Factor		
Prior concussion	1.34 (1.27-1.41)	1.15 (1.09-1.21)
Younger age (<40 y)	1.18 (1.16-1.20)	1.18 (1.16-1.21)
Male sex	1.10 (1.08-1.12)	1.13 (1.11-1.15)
Rural home	1.02 (0.99-1.04)	1.09 (1.07-1.12)
Diagnosis		
Alcohol misuse	4.68 (4.45-4.92)	2.70 (2.56-2.84)
Diabetes	1.42 (1.37-1.47)	1.48 (1.42-1.53)
Hypertension	0.95 (0.92-0.98)	0.93 (0.89-0.96)
Heart disease	1.12 (1.07-1.18)	1.02 (0.97-1.06)
Syncope	1.61 (1.57-1.66)	1.41 (1.37-1.45)
Sleep apnea	1.26 (1.21-1.30)	1.14 (1.10-1.18)
Osteoarthritis	1.26 (1.21-1.32)	1.24 (1.19-1.30)
Depression	2.54 (2.46-2.62)	1.78 (1.72-1.84)
Anxiety	2.21 (2.17-2.25)	2.00 (1.96-2.04)
Cancer	0.84 (0.79-0.89)	0.85 (0.80-0.90)
Socioeconomic^d^		
Highest	0.67 (0.65-0.69)	0.67 (0.65-0.70)
Next to highest	0.82 (0.79-0.84)	0.83 (0.81-0.86)
Next to lowest	1.36 (1.32-1.39)	1.33 (1.30-1.37)
Lowest	2.04 (1.99-2.09)	1.94 (1.89-1.99)
Crash time^e^		
Dawn	1.14 (1.09-1.18)	1.05 (1.01-1.10)
Morning	0.91 (0.89-0.93)	0.94 (0.92-0.97)
Afternoon	1.04 (1.02-1.07)	1.05 (1.03-1.08)
Night	1.13 (1.10-1.15)	1.09 (1.07-1.12)
Late night	1.46 (1.41-1.51)	1.29 (1.25-1.33)
Configuration^f^		
Single vehicle	1.35 (1.33-1.38)	1.06 (1.03-1.08)
Nonmotorized	1.16 (1.13-1.19)	0.78 (0.75-0.81)
Unlisted	1.21 (1.17-1.26)	0.89 (0.85-0.93)
Position^g^		
Passenger	1.32 (1.29-1.34)	1.30 (1.28-1.33)
Pedestrian	1.50 (1.47-1.53)	1.67 (1.62-1.72)
Acute care		
Ambulance	1.17 (1.15-1.19)	1.09 (1.07-1.11)
Triage severity	1.33 (1.30-1.35)	1.16 (1.14-1.19)
Injury severity^h^	1.03 (1.03-1.04)	1.02 (1.02-1.02)
Hospital admission	1.63 (1.57-1.68)	1.10 (1.06-1.15)

^a^ Estimates based on hazard ratio from Cox survival model.

^b^ No adjustments for baseline differences.

^c^ Adjusted for all other characteristics by regression model (Table 1, Table 2).

^d^ Referant is middle socioeconomic status.

^e^ Referant is evening interval.

^f^ Referant is multivehicle incident.

^g^ Referant is driver position.

^h^ Referent per unit increase in Injury Severity Score.

#### Adjusting for Confounding

**Figure 2.**
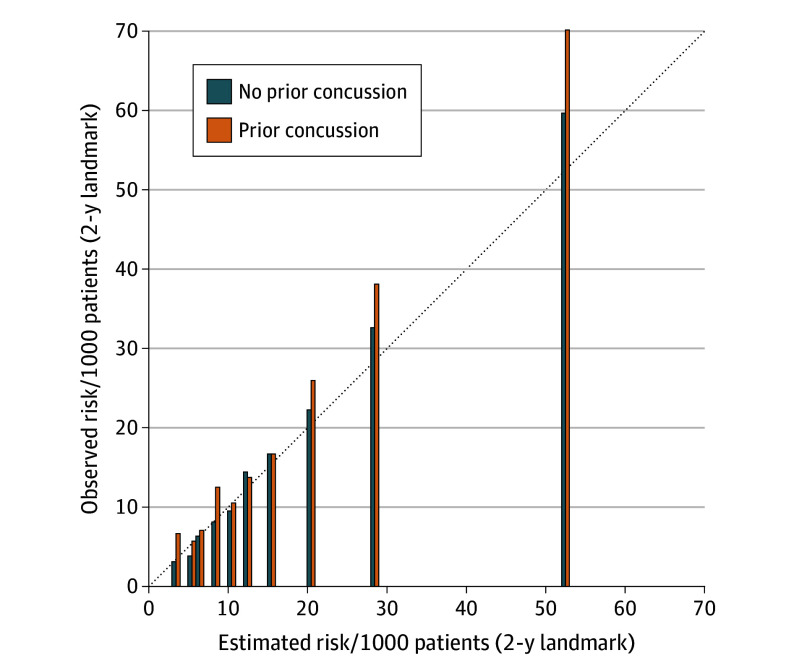
Risk at Different Predicted Probabilities The diagonal line represents the line of equivalence (ie, observed risk = predicted risk). Results show a wide range of estimated risk and generally higher relative risk for patients with a prior concussion; for example, top decile shows observed risk of 70.1 per 1000 for patients with a concussion and 59.6 per 1000 for patients with no prior concussion.

Further artificial intelligence techniques were applied to check robustness by adjusting for interactions, nonlinear relationships, and latent associations with unmeasured variables. Gradient boosting was tuned with a 0.03 learning rate, total of 750 iterations, weight of 3 for minimum child count, maximum tree depth of 4 levels, and typical run times of 15 minutes per model. The model fit was marginally improved (*C* statistic = 0.701 overall, 0.741 at 2-year landmark) and calibration was uneven (Figure 2). The model applied to patients who had a prior concussion estimated 409 expected cases of long-term disability (in contrast to 453 cases observed at 2 years). This contrast suggested a 28% relative increased risk of long-term disability for patients with a prior concussion (95% CI, 4%-52%; *P* = .02).

#### Clinical Distinctions

The increased risk of long-term disability after a concussion applied to diverse groups. Patients with lower socioeconomic status had a higher subsequent risk of long-term disability, yet the relative risk associated with a concussion applied at all levels of income (eTable 1 in Supplement 1). Similarly, those with higher crash severity had a higher subsequent risk of long-term disability, yet the relative risk associated with a concussion applied across the spectrum. Those with a relatively remote prior concussion and those with a relatively recent prior concussion each showed a significant increase in relative risk. Comparing patients with a single identified prior concussion to patients with multiple identified prior concussions showed no significant dose-response gradient. No subgroup analysis showed a significant opposite finding of reduced relative risk.

### Additional Outcomes

The increased risk of long-term disability after a concussion was distinct from other outcomes (eTable 2 in Supplement 1). A total of 37 385 patients died during follow-up, with no significant difference in risk per 1000 annually for patients with a prior concussion compared with patients with no prior concussion (3.25 per 1000 person-years vs 3.75 per 1000 person-years; *P* = .051). Similarly, a total of 322 290 patients were subsequently readmitted for any reason during follow-up, with no significant difference in risk per 1000 annually for those with a prior concussion (45.94 per 1000 person-years vs 43.25 per 1000 person-years; *P* = .13). Average short-term health care costs over the first year after the crash were also similar for those with and without a prior concussion ($4925 vs $4815; *P* = .41). In accord with past research, the annual risk of another future motor vehicle crash was increased for those with a prior concussion (21.74 per 1000 person-years vs 13.10 per 1000 person-years; *P* < .001).

## Discussion

We studied thousands of patients over many years and found a high risk of long-term disability after a motor vehicle crash, double the population norm. The major risk factors were demographic characteristics, alcohol misuse, psychiatric illnesses, and selected medical diseases. We also identified that a prior concussion in patients was associated with a worse risk of long-term disability after a motor vehicle crash. The overall effect size associated with a prior concussion was substantial, comparable with a diagnosis of sleep apnea, and greater than a diagnosis of heart disease. Together these findings suggest that recovery after a prior concussion can sometimes be incomplete and contributes to a possible loss of long-term resilience that impairs a person’s ability to return to full function after a motor vehicle crash occurring years later.

Our results support past research on long-term prognosis after a motor vehicle crash. A cohort analysis of 2019 adults after a noncatastrophic crash identified 23% were unable to resume full duties after 6 months.^9^ A cross-sectional survey of 442 adults following an earlier crash found multiple persisting health conditions limiting an individual’s ability to work.^40^ A cohort survey of 590 adults injured in a crash identified ongoing deficits due to pain, anxiety, mobility, and restricted activity more than 1 year later.^41^ Another prospective cohort study (64 007 patients) found 8% had a permanent medical impairment when assessed after 2 years.^42^ In addition, clinical studies often identify a high frequency of depression, social isolation, and reduced workforce participation among adults with a prior traumatic brain injury.^43^

### Limitations

An important limitation of our research is that each concussion is different, thereby making an exact prognosis hard to estimate due to lingering uncertainties.^44^ A randomized trial of concussions would be unthinkable, which means confounding can include genetics, lifestyle, personality, and many other unknowns. In our patients, for example, the available data lack specifics on how each prior concussion occurred, the severity of injury, pattern of symptoms, extent of comorbidities, and precise time for recovery.^45,46^ The data also lack records of childhood concussions; therefore, some patients in the control group may be misclassified and may have experienced head injuries decades earlier.^47^ Together, these uncertainties imply that clinical care needs to be personalized because aggregate statistics may underestimate the risk of long-term disability in some patients.^48^

A related set of limitations reflects the distinction between correlation and causality. Specifically, the observed data do not prove that a prior concussion directly contributed to the future disability or that preventing the concussion could have avoided the subsequent disability. In addition, the data do not identify whether a strategy of directly treating concussions symptoms would be more effective for patients than a general approach of encouraging greater traffic safety overall for reducing long-term disability.^49^ Moreover, a statistical model that adjusts for confounding by attributing risk to baseline characteristics may lead to overadjustment bias if a concussion can synergistically worsen a comorbidity (eg, a concussion exacerbating an underlying substance misuse disorder in a patient). These limitations are directions for future science.

Additional limitations relate to the amount of other information available and many further unknowns. We have no direct information on living circumstances, communication abilities, and other social determinants of disability.^50^ The unmeasured uncertainties also include details of the vehicle speed, reasons for travel, and total trips for each patient.^51^ We lack many clinical details about the initial treatment of the patient after the crash including subsequent outpatient services for community integration.^52,53^ Similarly, we lack engineering details about the conditions of the road, specifics of the vehicle, prevailing traffic enforcement, systems for safe transportation, and surrounding weather conditions.^54,55^ Collectively, these limitations might introduce substantial noise that generally bias analyses toward the null.

## Conclusions

This population-based cohort study suggests an increased patient risk of long-term disability when a prior concussion is followed later by a motor vehicle crash. This finding highlights the importance of counseling patients to reduce the risk of a motor vehicle crash.^43,49,56^ The finding also underlines the need to prevent head injuries in the first place by protective gear (eg, safety helmets) and preventive behaviors (eg, wearing seatbelts).^57,58^ The results might also encourage more concussion prevention for children and seniors even though these groups were not included in our study.^59,60^ A medication that could treat concussions and prevent disability, of course, is a topic for future research.^61,62,63^ In the interim, physicians caring for patients after a concussion should stress the importance of motor vehicle safety to reduce the risk of long-term disability.

## Notes

Supplement 1. eAppendix 1. Traffic crash code categorization eAppendix 2. Variable definitions and data sources eAppendix 3. Technical XGBoost details eFigure. Cohort selection eTable 1. Stratified analysis of long-term disability eTable 2. Other outcomes eTable 3. Propensity score test of robustness eTable 4. Estimates of standardized mean differences

Supplement 2. Data Sharing Statement
