# Supplementary material for: Prior Concussions and Risk of Disability for Patients After a Motor Vehicle Crash
Source: JAMA Netw Open. 2026 Jan 21;9(1):e2554831. doi: 10.1001/jamanetworkopen.2025.54831 (PMC12824782; doi:10.1001/jamanetworkopen.2025.54831)
Supplement: Supplement 1. — eAppendix 1. Traffic crash code categorization eAppendix 2. Variable definitions and data sources eAppendix 3. Technical XGBoost details eFigure. Cohort selection eTable 1. Stratified analysis of long-term disability eTable 2. Other outcomes eTable 3. Propensity score test of robustness eTable 4. Estimates of standardized mean differences [file jamanetwopen-e2554831-s001.pdf]

Supplemental Online Content

Redelmeier DA, Bhatt V, Drover SM. Prior concussions and risk of disability for patients after a motor vehicle crash. *JAMA Netw Open.* 2026;9(1):e2554831. doi:10.1001/jamanetworkopen.2025.54831

eAppendix 1. Traffic crash code categorization

eAppendix 2. Variable definitions and data sources

eAppendix 3. Technical XGBoost details

eFigure. Cohort selection

eTable 1. Stratified analysis of long-term disability

eTable 2. Other outcomes

eTable 3. Propensity score test of robustness

eTable 4. Estimates of standardized mean differences

This supplemental material has been provided by the authors to give readers additional information about their work.

© 2025 Redelmeier DA et al. *JAMA Network Open*.

eAppendix 1. Traffic Crash Code Categorization (using data from the National Ambulatory Care Reporting System [NACRS])

Configuration: Single/Multi-vehicle categorization. Single traffic crashes were defined as those that, according to emergency visit ICD-10 codes, involved only one motorized vehicle. Multi-vehicle crashes were defined as those that involved two or more motorized vehicles. For example, a traffic crash involving a cyclist (pedestrian) and a car would be counted as a single vehicle crash.

**Category**	**Summary of ICD-10 Codes Included**
Single	([V-0- | V-1- | V-6- | V-7- | V-8-] and not [V0-- | V1--])<br>*or*<br>([V--2 | V--3 | V--4 | V--5] and [V0-- | V1--])
Multiple	[V-2- | V-3- | V-4- | V-5- | V-9-]<br>and not [V-93 | V-98 | V-99 | V0-- | V1--]
Pedestrians Only	[V-0- | V-1- | V-6- | V-7- | V-8-] and [V0-- | V1--]
Unspecified	[V-93 | V-98 | V-99]

In the codes above, '-' indicates a wildcard digit (i.e., can be any value).

Configuration: Type of Vehicle or Transport. The type of vehicle or transport refers to the mode of transport used by the individual who presented at the emergency department, based on emergency visit ICD-10 codes.

**Category**	**Summary of ICD-10 Codes Included**
Pedestrians	[V0-- | V1--]
Motorcyclists	[V2-- | V3--]
Car	[V4--]
Pick-up Truck or Van	[V5--]
Heavy Transport	[V6--]

In the codes above, '-' indicates a wildcard digit (i.e., can be any value).

Configuration: Role. For individuals who were in or on a motorized vehicle, those with emergency visit ICD-10 codes specifying 'driver' were classified as such; otherwise, they were classified as a passenger. Pedestrians constituted a separate category.

© 2025 Redelmeier DA et al. *JAMA Network Open*.

Category	Summary of ICD-10 Codes Included
Driver	[V--0 | V--5 | V2-4 | V-94] and not [V2-5 | V-95 | V0-- | V1--]
Passenger	[V--1 | V--2 | V--3 | V--4 | V--6 | V--7 | V--8 | V--9 | V2-5 | V-95]<br>and not [V2-4 | V-94 | V0-- | V1--]
Pedestrian	[V0-- | V1--]

In the codes above, '-' indicates a wildcard digit (i.e., can be any value).

Resources that informed traffic crash categorization.

International Statistical Classification of Diseases and Related Health Problems 10th Revision (ICD-10)-WHO Version for ;2016, Transport Accidents (V01-V99)

ICD-10 Version:2016 (who.int)

Association of Public Health Epidemiologists in Ontario, Recommended ICD-10-CA Codes for Injury Core Indicators

APHEO - 10 Recommended ICD-10-CA Codes for Injury Indicators

© 2025 Redelmeier DA et al. *JAMA Network Open*.

eAppendix 2. Variable Definitions and Data Sources

Exposure Codes

Exposure	OHIP Diagnostic code
Acute concussion	850

OHIP: Ontario Health Insurance Plan

Outcome Codes

Exposure	OHIP Fee code
Long-term disability	K050-K054, K057-K060

Baseline Characteristic Codes

Condition	OHIP Diagnostic code
Alcohol use	303
Ankle sprain (5 year lookback)	845
Anxiety	300
Cancer	140 to 208
Depression	311
Diabetes mellitus	250
Heart disease	410-429
Hypertension	401
Osteoarthritis	715
Sleep apnea	786
Syncope	780

© 2025 Redelmeier DA et al. *JAMA Network Open*.

ICES Data Sources Used in this Study

Database Name	Description	Use in this Study
**Discharge Abstract Database (DAD)**	Contains data on inpatient hospital discharges (inpatient acute, chronic, rehab) and day surgeries.[1]	• Hospital admission details following traffic crash
**National Ambulatory Care Reporting System (NACRS)**	Captures information on patient visits to hospital and community based ambulatory care, including day surgery, outpatient clinics, and emergency departments.[2]	• Identification and details of visits to emergency departments for traffic crash<br>• Secondary outcomes
**Ontario Health Insurance Plan (OHIP)**	Contains claims paid for by the Ontario Health Insurance Plan: these data cover all health care providers who can claim under OHIP.[3]	• Prior concussion<br>• Baseline (past) diagnoses<br>• Long-term disability
**Postal Code Conversion File (PCCF)**	Conversion of Canadian postal code to StatsCan standard geographic areas.[4]	• Used in conjunction with RPDB to identify neighbourhood-level socioeconomic characteristics
**Registered Persons Database (RPDB)**	Provides basic demographic information (age, sex, location of residence, date of birth, date of death if applicable) about anyone who has ever received an Ontario health card number.[5]	• Baseline demographic and socioeconomic characteristics
**Data sources used in health care cost computation***	The ICES GETCOST SAS macro computes individual-level health care costs for requested time periods.[6]	• Healthcare costs in the first year after traffic crash (secondary outcome)

© 2025 Redelmeier DA et al. *JAMA Network Open*.

Footnotes

* List of ICES datasets used by the ICES GETCOST SAS macro:

- Assistive Devices Program (ADP)
- Client Agency Program Enrolment (CAPE)
- Discharge Abstract Database (DAD)
- Continuing Care Reporting System (CCRS)
- Estimated Schedule of Benefits (SOB) price associated with each OHIP fee code and suffix (ESTSOB)
- GAPP Decision Support Systems (Physician Payments; GAPP)
- Home Care Database (HCD)
- INST: Information about Ontario health care institutions funded by the Ministry of Health and Long-Term Care
- National Ambulatory Care Reporting System (NACRS)
- New Drug Funding Program (NDFP)
- National Rehabilitation Reporting System (NRS)
- Ontario Case Costing Initiative (OCCI)
- Ontario Drug Benefit Claims (ODB)
- Ontario Home Care Administrative System (OHCAS)
- Ontario Health Insurance Plan (OHIP)
- Ontario Mental Health Reporting System (OMHRS)
- Same Day Surgery Database (SDS)

References

1. Juurlink DN, Preyra C, Croxford R, Chong A, Austin P, Tu J, et al. Canadian Institute for Health Information Discharge Abstract Database: A Validation Study. Toronto (ON): Institute for Clinical Evaluative Sciences; 2006. (ICES | Canadian Institute for Health Information Discharge Abstract Database: A Validation Study)
2. Canadian Institute for Health Information. National Ambulatory Care Reporting System (NACRS) metadata. Accessed August 1, 2025 (National Ambulatory Care Reporting System (NACRS) metadata | CIHI).
3. Government of Ontario, Ontario Ministry of Health. OHIP Schedule of Benefits and fees. Accessed August 1, 2025 (OHIP Schedule of Benefits and fees | ontario.ca).
4. Statistics Canada. Postal Code[OM] Conversion File Plus (Catalogue no. 82F0086X). Accessed August 1, 2025 (Postal Code OM Conversion File Plus (PCCF+)).
5. Government of Ontario, Ontario Ministry of Health. Registered Persons Database (RPDB). Ontario Data Catalogue. Accessed August 1, 2025 (Registered Persons Database (RPDB) - Dataset - Ontario Data Catalogue).
6. Wodchis WP, Bushmeneva K, Nikitovic M, McKillop I. Guidelines on Person-Level Costing Using Administrative Databases in Ontario. Working Paper Series. Vol 1. Toronto: Health System Performance Research Network; 2013.

© 2025 Redelmeier DA et al. *JAMA Network Open*.

eAppendix 3. Technical XGBoost details

An XGBoost-powered cox regression model, a machine learning method, was used to predict the hazard ratio (HR) and 2-year risk of long-term disability. The XGBoost cox regression model was trained on a random sample of the control (no remote concussion) cohort (80% sample, N = 710,506) and was validated on the remainder of the sprain cohort (20% sample, N = 177,627), truncating follow-up at 2 years for both the train and test set. After validating the machine learning model in the control (no remote concussion) cohort, it was applied separately to the full remote concussion cohort (N = 19,851).

The XGBoost-predicted 2-year risk for each individual in the test set was estimated as:

2-year risk ≈ Mean baseline cumulative hazard in the training set · Predicted HR in the test set

Harrell's Concordance Index was estimated by first fitting a cox model with the 2-year XGBoost-estimated risk as the predictor and time to crash (truncated at 2 years follow-up) as the outcome. The Harrell's Concordance Index (C-index) of this model estimates the C-index of the XGBoost cox regression model.

Predictors of long-term disability following traffic crash in the machine learning model included: age, binary sex, rural/urban residence, income quintile, alcohol misuse, diabetes, hypertension, heart disease, unexplained syncope, sleep apnea, osteoarthritis, depression, anxiety, cancer, and traffic crash features (ambulance transport, triage level, hospital admission, crash time, number of vehicles involved, role [passenger, driver, pedestrian], and injury severity score).

In addition to the landmark 2-year analysis, XGBoost analyses truncating follow-up at 1, 5, and 10 years were explored following the same methods as described above. The only difference across these models were some XGBoost parameters (see below), which were modulated to balance overfitting versus underfitting for each follow-up time. Results were similar across timepoints.

To implement the gradient boosting model, the package XGBoost version 1.4.0.1 was used on R version 3.6.1. The parameters of the model are listed below (any parameters not specified below were set at the default for R package XGBoost version 1.4.0.1). The run time for fitting each gradient boosting model and producing predicted probabilities was approximately 15 minutes per model.

**XGBoost parameter (description)**	**1 year**	**2 year***	**5 year**	**10 year**
eta (learning rate)	0.03	**0.03**	0.01	0.01
max_depth (tree depth)	4	**4**	3	3
Gamma (regularization parameter)	0	**0**	3	3
min_child_weight (minimum number of instance weights in a leaf node)	3	**3**	8	8
Lambda (L2 regularization term on leaf weights)	0	**0**	7	7
Alpha (L1 regularization term on leaf weights)	0	**0**	2	2
nrounds (number of iterations)	750	**750**	750	100

*2 year was the landmark analysis

eFigure. Cohort Selection

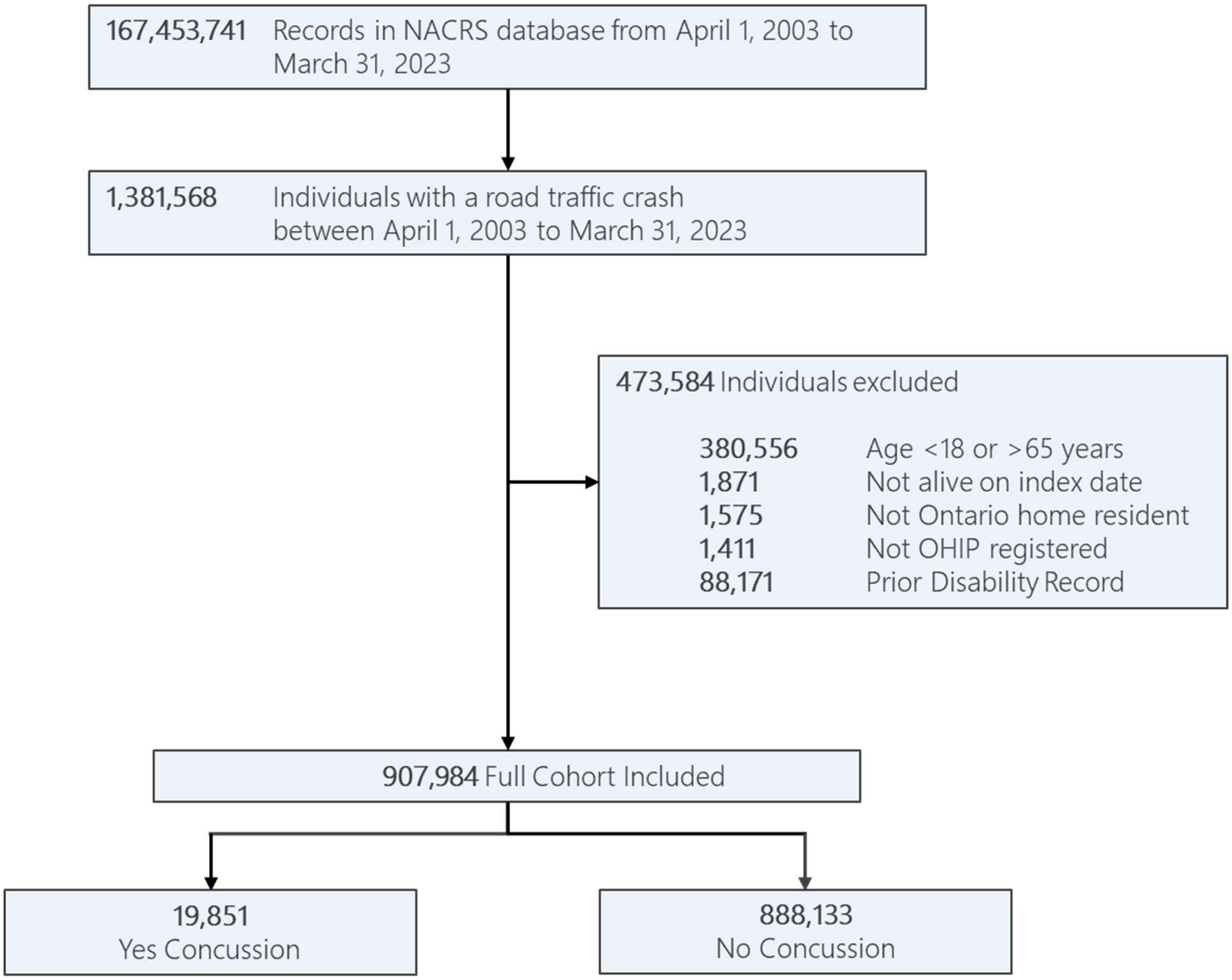

Index date refers to the NACRS date of visit for the first road traffic crash for each individual within the accrual window.

Concussion exposure is defined as concussion in the 5 years prior to the index crash date.

© 2025 Redelmeier DA et al. *JAMA Network Open*.

eTable 1. Stratified Analysis of Long-term Disability

Analysis	Total Events	Remote Concussion Events	Remote Concussion Patients	Remote Concussion Patient-Years	Remote Concussion Rate*	Control (No remote concussion) Events	Control (No remote concussion) Patients	Control (No remote concussion) Patient-Years	Control (No remote concussion) Rate*	Hazard Ratio †	Confidence Interval	P-value
**Primary Analysis**												
Long-term disability	54,678	1,311	19,851	160,787	**8.15**	53,367	888,133	9,382,718	**5.69**	**1.34**	1.27 to 1.41	0.000
**Demographic Subgroup**												
18 - 39 years	34,145	1,031	15,185	122,210	**8.44**	33,114	513,565	5,481,393	**6.04**	**1.31**	1.23 to 1.40	0.000
40 - 65 years	20,533	280	4,666	38,577	**7.26**	20,253	374,568	3,901,325	**5.19**	**1.30**	1.15 to 1.46	0.000
Female	25,252	591	9,962	74,823	**7.90**	24,661	425,587	4,559,711	**5.41**	**1.33**	1.22 to 1.44	0.000
Male	29,426	720	9,889	85,964	**8.38**	28,706	462,546	4,823,007	**5.95**	**1.35**	1.25 to 1.45	0.000
Urban home	47,683	1,116	16,891	134,924	**8.27**	46,567	781,009	8,186,135	**5.69**	**1.35**	1.27 to 1.44	0.000
Rural home	6,995	195	2,960	25,863	**7.54**	6,800	107,124	1,196,584	**5.68**	**1.26**	1.09 to 1.45	0.002
**Socioeconomic Subgroup**												
Highest	5,566	178	4,072	32,865	**5.42**	5,388	156,359	1,691,720	**3.18**	**1.61**	1.38 to 1.87	0.000
Next to highest	7,659	187	4,142	34,925	**5.35**	7,472	177,179	1,919,410	**3.89**	**1.30**	1.12 to 1.50	0.000
Middle	9,533	234	3,920	31,457	**7.44**	9,299	182,936	1,947,390	**4.78**	**1.46**	1.28 to 1.66	0.000
Next to lowest	12,882	309	3,842	30,897	**10.00**	12,573	184,650	1,936,333	**6.49**	**1.44**	1.29 to 1.61	0.000
Lowest	19,038	403	3,875	30,643	**13.15**	18,635	187,009	1,887,865	**9.87**	**1.24**	1.12 to 1.37	0.000
**Prior Concussion**												
Remote (≥ 2 years)	54,046	679	10,464	86,292	**7.87**	53,367	888,133	9,382,718	**5.69**	**1.30**	1.21 to 1.41	0.000
Recent (< 2 years)	53,999	632	9,387	74,495	**8.48**	53,367	888,133	9,382,718	**5.69**	**1.38**	1.27 to 1.49	0.000
Multiple (≥ 2 identified)	53,444	77	1,567	9,608	**8.01**	53,367	888,133	9,382,718	**5.69**	**1.20**	0.96 to 1.50	0.106
Single (1 identified)	54,601	1,234	18,284	151,179	**8.16**	53,367	888,133	9,382,718	**5.69**	**1.35**	1.27 to 1.43	0.000
**Crash Severity**												
Ambulance - no	28,455	744	12,127	96,345	**7.72**	27,711	497,944	5,248,102	**5.28**	**1.38**	1.28 to 1.48	0.000
Ambulance - yes	26,223	567	7,724	64,442	**8.80**	25,656	390,189	4,134,616	**6.21**	**1.32**	1.21 to 1.43	0.000
Triage severity - lower	41,260	954	15,221	126,987	**7.51**	40,306	699,846	7,592,282	**5.31**	**1.33**	1.25 to 1.42	0.000
Triage severity- higher	13,418	357	4,630	33,800	**10.56**	13,061	188,287	1,790,436	**7.29**	**1.32**	1.18 to 1.46	0.000
Hospital admission - no	51,234	1,240	19,248	155,660	**7.97**	49,994	851,142	9,007,347	**5.55**	**1.34**	1.27 to 1.42	0.000
Hospital admission - yes	3,444	71	603	5,127	**13.85**	3,373	36,991	375,371	**8.99**	**1.43**	1.13 to 1.81	0.003

© 2025 Redelmeier DA et al. *JAMA Network Open.*

eTable 2. Other outcomes

Analysis	Total Events	Remote Concussion Events	Remote Concussion Patients	Remote Concussion Patient-Years	Remote Concussion Rate*	Control (No remote concussion) Events	Control (No remote concussion) Patients	Control (No remote concussion) Patient-Years	Control (No remote concussion) Rate*	Hazard Ratio †	Confidence Interval	P-value
**Primary Analysis**												
Long-term disability	54,678	1,311	19,851	160,787	**8.15**	53,367	888,133	9,382,718	**5.69**	**1.34**	1.27 to 1.41	< 0.001
**Other Outcomes #**												
All-cause mortality	37,385	555	19,851	170,876	**3.25**	36,830	888,133	9,828,856	**3.75**	**0.92**	0.85 to 1.00	0.051
Hospitalization for any cause	322,290	5,980	19,851	130,158	**45.94**	316,310	888,133	7,313,936	**43.25**	**1.02**	0.99 to 1.05	0.133
Another traffic crash	118,679	3,202	19,851	147,279	**21.74**	115,477	888,133	8,876,114	**13.01**	**1.53**	1.48 to 1.58	0.000

Healthcare Costs ¶	Total Cost Overall	Remote Concussion Total Cost	Remote Concussion Patients	Remote Concussion Patient-Years	Remote Concussion Average Total Cost	Control (No remote concussion) Total Cost	Control (No remote concussion) Patients	Control (No remote concussion) Patient-Years	Control (No remote concussion) Average Total Cost	Average Cost Difference	Confidence Interval	P-Value
First year after traffic crash	4,374,354,904	97,764,254	19,851	19,796	**4924.90**	4,276,590,650	888,133	885,214	**4815.26**	**109.64**	-151.72 to 371.00	0.411

* risk is rate per thousand person-years

† calculated based on hazard rates without adjustments for covariates

# Refer to Appendix B for ICD-10 codes

¶ total cost standardized to 2023 calculated using GETCOST macro at ICES (see Guidelines on Person- Level Costing Using Administrative Databases in Ontario. Available online: https://tspace.library.utoronto.ca/bitstream/1807/87373/1/Wodchis%20et%20al_2013_Guidelines%20on%20Person-Level%20Costing.pdf). Total cost was modelled using a linear regression

© 2025 Redelmeier DA et al. *JAMA Network Open.*

eTable 3. Propensity Score Test of Robustness

Analysis	Total Events	Remote Concussion				Control (No remote concussion)				Hazard Ratio †	Confidence Interval	P-value
		Events	Patients	Patient-Years	Rate*	Events	Patients	Patient-Years	Rate*			
**Primary Analysis**												
Long-term disability	54,678	1,311	19,851	160,787	**8.15**	53,367	888,133	9,382,718	**5.69**	**1.34**	1.27 to 1.41	< 0.001
**Adjusted Analysis**												
Long-term disability	54,678	1,311	19,851	160,787	**--**	53,367	888,133	9,382,718	**--**	**1.15**	1.09 to 1.21	< 0.001
**Test of Robustness #**												
Propensity score matching	2,718	1,310	19,850	160,787	**8.15**	1,408	19,850	211,715	**6.65**	**1.14**	1.06 to 1.23	< 0.001

* risk is rate per thousand person-years

† calculated based on hazard rates without adjustments for covariates

# Based on matching features in Table 1 of Baseline Characteristics. For matching, age was modelled continuously using a restricted cubic spline. A greedy nearest-neighbor matching algorithm with a 1:1 matching ratio and a caliper of 0.2 standard deviations of the logit of the propensity score was applied.

© 2025 Redelmeier DA et al. *JAMA Network Open*.

eTable 4. Estimates of Standardized Mean Differences

Baseline Patient Characteristics

		Prior Concussion		Standardized
		Yes	No	Mean Difference †
		(n = 19,851)	(n = 888,133)	
DEMOGRAPHIC DATA				
Age (years)	18-39	15,185 (76.5%)	513,565 (57.8%)	0.406
	40-65	4,666 (23.5%)	374,568 (42.2%)	
Sex	Male	9,889 (49.8%)	462,546 (52.1%)	0.045
	Female	9,962 (50.2%)	425,587 (47.9%)	
Home	Urban	16,891 (85.1%)	781,009 (87.9%)	0.083
	Rural	2,960 (14.9%)	107,124 (12.1%)	
PAST DIAGNOSES #				
Alcohol misuse	Yes	303 (1.5%)	6,952 (0.8%)	0.070
Diabetes	Yes	578 (2.9%)	42,350 (4.8%)	0.097
Hypertension	Yes	764 (3.8%)	59,760 (6.7%)	0.129
Heart disease	Yes	668 (3.4%)	30,433 (3.4%)	0.003
Syncope	Yes	2,350 (11.8%)	54,459 (6.1%)	0.201
Sleep apnea	Yes	1,232 (6.2%)	42,525 (4.8%)	0.062
Osteoarthritis	Yes	525 (2.6%)	28,904 (3.3%)	0.036
Depression	Yes	1,395 (7.0%)	31,288 (3.5%)	0.157
Anxiety	Yes	4,979 (25.1%)	139,607 (15.7%)	0.234
Cancer	Yes	464 (2.3%)	23,544 (2.7%)	0.020
SOCIOECONOMIC STATUS *				
Level	Highest	4,072 (20.5%)	156,359 (17.6%)	0.074
	Next Highest	4,142 (20.9%)	177,179 (19.9%)	0.023
	Middle	3,920 (19.7%)	182,936 (20.6%)	0.021
	Next Lowest	3,842 (19.4%)	184,650 (20.8%)	0.036
	Lowest	3,875 (19.5%)	187,009 (21.1%)	0.038

Footnotes
# based on previous year
* based on home neighbourhood, missing data coded as lowest
† based on pooled standard deviation

© 2025 Redelmeier DA et al. *JAMA Network Open.*
