# Supplementary material for: Prior Concussions and Risk of Disability for Patients After a Motor Vehicle Crash
Source: JAMA Netw Open. 2026 Jan 21;9(1):e2554831. doi: 10.1001/jamanetworkopen.2025.54831 (PMC12824782; doi:10.1001/jamanetworkopen.2025.54831)
Supplement: Supplement 2. — Data Sharing Statement [file jamanetwopen-e2554831-s002.pdf]

Data Sharing Statement

Redelmeier. Prior Concussions and Risk of Disability for Patients After a Motor Vehicle Crash. *JAMA Netw Open*. Published January 21, 2026. doi:10.1001/jamanetworkopen.2025.54831

Data
Data available: No

Additional Information
Explanation for why data not available: The study dataset is held securely in coded form at ICES. While legal data sharing agreements between ICES and data providers (eg, healthcare organizations and government) prohibit ICES from making the dataset publicly available, access may be granted to investigators who meet criteria for confidential access, available at www.ices.on.ca/DAS (email das@ices.on.ca). The full dataset creation plan and analytic code are available from the authors upon request, understanding that the computer programs might rely upon coding templates or macros unique to ICES.
